# Entrepreneurial role models and college students’ entrepreneurial calling: A moderated mediation model

**DOI:** 10.3389/fpsyg.2023.1129495

**Published:** 2023-02-22

**Authors:** Dongmei Jin, Xiaomeng Liu, Fuqin Zhang, Zhiyi Wen

**Affiliations:** ^1^Business School, Beijing Technology and Business University, Beijing, China; ^2^Personnel Testing Authorities, Ministry of Human Resources and Social Security of the PRC, Beijing, China

**Keywords:** entrepreneurial role models, entrepreneurial calling, entrepreneurial perceived behavioral control, entrepreneurial hands-on practice, entrepreneurship education

## Abstract

**Introduction:**

College entrepreneurship education should not only cultivate a group of college students who have strong willingness to start a business immediately after graduation, but also pay attention to future entrepreneurship success of college students. Correspondingly, in addition to attaching importance to improving college students’ entrepreneurial intention, college entrepreneurship education should pay attention to improving college students’ entrepreneurial calling. At present, there is insufficient research on the association between entrepreneurial role models and entrepreneurial calling. We aim to study the mechanism and boundary condition of the association between entrepreneurial role models and entrepreneurial calling.

**Methods:**

A longitudinal survey was distributed among 519 students from 16 colleges and universities in China. In the survey, the college students answered questions on entrepreneurial role models, entrepreneurial calling, entrepreneurial perceived behavioral control and entrepreneurial hands-on practice. Hierarchical regression was conducted, testing the association between entrepreneurial role models and entrepreneurial calling of college students, mediated by entrepreneurial perceived behavioral control and moderated by entrepreneurial hands-on practice.

**Results:**

Therefore, based on the social learning theory, the theory of planned behavior and the entrepreneurial event model, and by hierarchical regression of the data, this study confirmed that entrepreneurial role models were positively associated with college students’ entrepreneurial calling by partially mediating with entrepreneurial perceived behavioral control. Moreover, Entrepreneurial hands-on practice positively moderated not only the relationship between entrepreneurial perceived behavioral control and entrepreneurial calling, but also the mediating association of entrepreneurial perceived behavioral control between entrepreneurial role models and entrepreneurial calling.

**Discussion:**

This study not only enriches the theoretical research on entrepreneurial calling and entrepreneurial role models, but also provides valuable educational enlightenment for colleges and universities to improve the students’ entrepreneurial calling.

## 1. Introduction

Entrepreneurship can not only boost the national economic growth, promote the transformation of the industrial structure and the conversion of the old and new kinetic energy, but also create jobs and raise the level of the social employment. In 2014, the Chinese government called for mass innovation and mass entrepreneurship. Under the influence of the current internal and external economic environment and the entrepreneurship policy in China, college students have become the new main body of entrepreneurship (Kong et al., 2020). College entrepreneurship education should not only cultivate a group of college students who have strong willingness to start a business immediately after graduation, but also pay attention to future entrepreneurship success of college students (Franco et al., 2010). Correspondingly, in addition to attaching importance to improving college students’ entrepreneurial intention, college entrepreneurship education should pay attention to improving college students’ meaningful passion for entrepreneurship, that is entrepreneurial calling. If the entrepreneurial calling level of college students is improved, a number of them will hold the dream of entrepreneurship, constantly improve entrepreneurial ability, develop entrepreneurial resources, and when the time is ripe, they will have the entrepreneurial intention to start a business (Duffy and Sedlacek, 2007; Dik and Steger, 2008; Douglass and Duffy, 2015; Hirschi et al., 2018; Lau et al., 2020; Luo M. et al., 2022). In the long run, the college entrepreneurship education emphasizing on cultivating entrepreneurial calling can effectively improve the number of college graduates who will set up their own business and succeed in entrepreneurship in the future, so it has strong practical significance and potential value.

The innovation and entrepreneurship education in colleges and universities will become an obvious trend in the future (Qu, 2021). Entrepreneurship education can impart entrepreneurial knowledge (Low, 2001; Boldureanu et al., 2020; Lee et al., 2021), skills (Nian et al., 2014; Boldureanu et al., 2020; Lee et al., 2021; Soomro and Shah, 2022) and attitudes (Duong, 2021; Otache et al., 2021). It also can cultivate entrepreneurs (Volery and Mueller, 2006; Woollard et al., 2007). College entrepreneurship education can enhance college students’ entrepreneurial creativity (Wang C. et al., 2022), entrepreneurial process competence (Mets et al., 2022), entrepreneurial capability (Yin and Wang, 2017), business opportunity recognition (Othman et al., 2020), entrepreneurial self-efficacy (Luo L. et al., 2022; Wu et al., 2022), entrepreneurial alertness (Saadat et al., 2021), entrepreneurial intention (Mónico et al., 2021; Anwar et al., 2022; Hieu and Loan, 2022; Otache et al., 2022), entrepreneurial mindset (Jiatong et al., 2021), entrepreneurial preparation (Saptono et al., 2020), entrepreneurial passion (Lee et al., 2021; Uddin et al., 2022) and entrepreneurial behaviors (Cai et al., 2021). College entrepreneurship education should be open. The entrepreneurial activities of college students can be developed by the school or by the students themselves. The exploration of self-employment of college students should also be included in the entrepreneurial education system of colleges and universities. The entrepreneurial role models of college students can be set and publicized by colleges and universities or spontaneously concerned by students.

Entrepreneurial role model is a way of entrepreneurial education and an important factor affecting college students’ career exploration (Luo M. et al., 2022). At present, there is insufficient research on the association between entrepreneurial role models and entrepreneurial calling, which prompts us to conduct empirical research. This study specifically explored the mechanism of the association between entrepreneurial role models and entrepreneurial calling, and examined the mediating role of entrepreneurial perceived behavioral control and the moderating role of entrepreneurial hands-on practice.

## 2. Theoretical foundation and hypotheses

### 2.1. Entrepreneurial calling and entrepreneurial role models

Calling is the continuous meaningful passion of an individual for a particular occupation (Dobrow and Tosti-Kharas, 2011). It is influenced by factors such as individual ability, behavioral participation and social comfort in the field where calling is directed (Ericsson et al., 1993; Winner, 2000; Dobrow, 2013). Entrepreneurship is an occupation of identifying, evaluating and developing opportunities to create future goods and services (Venkataraman, 2012). Entrepreneurial calling was proposed by Da Palma et al. (2018) and Tian et al. (2018), which was a calling that takes entrepreneurship as an occupation. Entrepreneurial calling is different from entrepreneurial intention and entrepreneurial passion. Entrepreneurial intention is an individual’s tendency to start a business when choosing an occupation, and is considered to be a proximal and immediate predictor of entrepreneurial behavior (Bird and Jelinek, 1988; Bagozzi et al., 1989; Krueger et al., 2000; Majeed et al., 2021). Entrepreneurial passion is the passion experienced by individuals in the entrepreneurial circumstances (Mageau et al., 2009; Perrewe et al., 2013; Cardon and Kirk, 2015; Türk et al., 2020; Yi et al., 2020; Bignetti et al., 2021; Liu et al., 2021). According to the stage of entrepreneurial development, entrepreneurial passion can be divided into the passion for founding enterprises, the passion for discovering opportunities and the passion for developing enterprises (Cardon et al., 2013). Entrepreneurial passion can be generated in real or simulated entrepreneurial circumstances. According to Dobrow (2013) research on calling, entrepreneurial calling is a continuous single-dimensional construct. It is strong and lasting positive emotional experience about entrepreneurship, based on the identification of the meaning and value of entrepreneurship and the identity of entrepreneurs, accompanied by entrepreneurial desire. When individuals are not inclined to choose entrepreneurship as a career, or are not in the entrepreneurial situation, they may have entrepreneurial calling. That is to say, individuals may have entrepreneurial calling when they have no entrepreneurial intention or entrepreneurial passion. Previous studies showed that entrepreneurial calling of college students was positively associated with their entrepreneurial ability (Douglass and Duffy, 2015) and future entrepreneurial choice (Duffy and Sedlacek, 2007; Dik and Steger, 2008).

Shapero and Sokol (1982) were the first to introduce role models into the entrepreneurship research. Entrepreneurial role models are the ones who inspire and motivate people to engage in the entrepreneurial activities (Maziriri et al., 2019). They provide spiritual stimulation or behavioral guidance to people in the field of entrepreneurship, and their behaviors and personal styles are imitated by others (Bandura, 1986; Gibson, 2004; Bosma et al., 2012). Previous studies showed that entrepreneurial role models were positively associated with college students’ entrepreneurial attitude (Nowinski and Haddoud, 2019), perception of entrepreneurial feasibility (Van Trang et al., 2019), entrepreneurial perceived behavioral control (Fellnhofer, 2017a; Fellnhofer and Mueller, 2018), entrepreneurial passion (Fellnhofer, 2017b) and entrepreneurial intention (Liu et al., 2005; Kong et al., 2020; Luo M. et al., 2022).

Bandura (1977a) social learning theory holds that in social situations, individuals can learn by observing other people’s behaviors and their results. The occurrence of social learning is conditional. The conditions include the attraction, reputation or authority of role models, and the need satisfaction of learners (Bandura, 1986). At present, the Chinese government and society advocate entrepreneurship strongly. Successful entrepreneurs have high social prestige and are easy to be imitated. Meanwhile, college students are in the stage of career exploration and are easily attracted by careers that can meet their needs of autonomy, competence and social connection, such as entrepreneurship (Al-Jubari et al., 2018). Therefore, it will be possible for college students to observe and imitate the entrepreneurs as role models. Moreover, the social learning theory holds that individuals’ observation and perception of the role models’ behaviors or psychology, reinforced by the positive consequences, will stimulate their expectation of the same consequences if they adopt the same behaviors and psychology (Bandura, 1977a).

According to the social learning theory, this study held that in the process of observing and learning from entrepreneurial role models, college students’ entrepreneurial calling would be enhanced. First of all, entrepreneurial role models would enhance the positive emotion of college students about entrepreneurship. Passion for entrepreneurship is the defining characteristic of entrepreneurial role models (Chen et al., 2009; Cardon et al., 2013; Saboor et al., 2020). In the process of contacting entrepreneurial role models, college students will be infected by the positive and strong emotion of entrepreneurial role models. Emotional contagion is an emotional experience caused by and matched with the emotions of others (Morris and Feldman, 1996). Emotional contagion has two channels: primitive emotional contagion and conscious emotional contagion (Barsade, 2002). Primitive emotional contagion is generated automatically in the sub-consciousness (Hatfield et al., 1992). The process of conscious emotional contagion is participated by consciousness (Barsade, 2002; Hennig-Thurau et al., 2006). According to the social learning theory, the process of college students’ being infected by the original emotion of entrepreneurial role models is that they automatically and unconsciously imitate the language, facial expression and behavior of entrepreneurial role models. In addition, emotional experience matching the entrepreneurial role model’s love for entrepreneurship is generated in the process of changes in their own language, expression and action (Hatfield et al., 1992). The process of college students’ being affected consciously by the emotions of entrepreneurial role models is that they transfer entrepreneurial role models’ positive emotions towards entrepreneurship into of their own after observing that the positive and strong emotions of entrepreneurial role models are positively strengthened by entrepreneurial results (Falkenberg et al., 2008). Secondly, entrepreneurial role models could enhance college students’ identification of significance and value of entrepreneurship and their identity of entrepreneurs. Entrepreneurial role models have rich entrepreneurial experience and perception. Through words and deeds, they express their strong identification with the entrepreneurial value and entrepreneur identity which were observed or felt by college students. The entrepreneurial achievements made by entrepreneurial role models are positive alternative results for college students. In their view, the salient characteristics of entrepreneurs such as entrepreneurial value identification and entrepreneur identity are reinforced by positive alternative results, so they will have high expectations for these characteristics and then imitate them (Bandura, 1987). The empirical studies showed that entrepreneurial role models were positively associated with individuals’ professional values (Pablo-Lerchundi et al., 2015) and their identity with entrepreneurs (Gibson, 2004; Boldureanu et al., 2020). Lastly, entrepreneurial role models could enhance the entrepreneurial desire of college students. In the process of observing and imitating entrepreneurial role models, college students’ positive emotion towards entrepreneurship, identification with the entrepreneurial value and entrepreneur identity are enhanced. Moreover, entrepreneurship can meet the advantage needs of college students, such as autonomy, competence and social connection (Al-Jubari et al., 2018), therefore their desire for entrepreneurship will be strengthened. Previous empirical studies have also shown that entrepreneurial role models have significant positive association with college students’ aspirations of entrepreneurship (Meccheri and Pelloni, 2006). In summary, in the process of observing and learning from entrepreneurial role models, college students’ positive emotion towards entrepreneurship would be enhanced; the cognitive basis of this emotion – the identification of the meaning and value of entrepreneurship and the identity of entrepreneurs would be enhanced; and the desire to start a business that accompanies this emotion would be enhanced. Therefore, the following hypothesis was proposed in this study:

*H1*: Entrepreneurial role models are positively associated with college students’ entrepreneurial calling.

### 2.2. The mediating role of entrepreneurial perceived behavioral control

Ajzen’s theory of planned behavior and Shapero’s model of entrepreneurial event are the two most promising approaches to explaining the decision to establish a new business firm in the future (Franco et al., 2010). The theory of planned behavior and the entrepreneurial event model are considered highly analogous to each other while perceived behavioral control and perceived feasibility of an entrepreneurial idea are elements conceptually associated with perceived self-efficacy (Krueger et al., 2000). Some studies utilized both the theory of planned behavior and the entrepreneurial event model to determine the main antecedents of university students’ entrepreneurial intention (Krueger et al., 2000; Veciana et al., 2005; Liñán et al., 2011; Zhang et al., 2014; Celik et al., 2021). According to Shapero’s model of entrepreneurial event, perceived entrepreneurial desire, perceived entrepreneurial feasibility and propensity to act are the three main antecedents of an individual’s entrepreneurial intention (Shapero and Sokol, 1982; Peterman and Kennedy, 2003). According to the theory of planned behavior proposed by Ajzen (1991), the three preconditions of behavioral intention are attitude toward behavior, subjective norms and perceived behavioral control. Among them, perceived behavioral control is an important concept in the theory of planned behavior, which has been widely used as an independent concept (Ajzen, 1991). Perceived behavioral control refers to an individual’s belief about the possibility and difficulty of the particular behavior (Bandura, 1977b; Ajzen, 1991). Engle et al. (2010) and Yean et al. (2015) believed that individuals had various beliefs related to activities, thus generating the perception of whether the activities are successful. Perceived behavioral control includes not only the perception of competence reflected by self-efficacy (Bandura et al., 1980; Ajzen, 2002), but also the perception of the resources needed to produce the behavior (Büssing et al., 2019). Although conceptually it is considered to share some degree of similarity to self-efficacy (Bandura, 1977b) and perceived feasibility (Shapero and Sokol, 1982), it is widely used as a stand-alone concept because it encompasses not only the feeling of being able but also the perception about the controllability of the behavior (Liñán and Chen, 2009).

Entrepreneurial perceived behavioral control refers to the subjective evaluation of a person’s own entrepreneurial ability, resources, and the possibility of entrepreneurial success (Yang, 2013). The level of entrepreneurial perceived behavioral control is sensitive to environmental factors like cultural influences (Krueger et al., 2000), social pressures (Liñán and Chen, 2009), competitive environments (Anggadwita and Mustafid, 2014), or governmental support (Anggadwita and Dhewanto, 2016). Entrepreneurial perceived behavioral control mediates the relationship between entrepreneurship education (Yurtkorua et al., 2014; Duong, 2021; Aga and Singh, 2022), access to finance, university environment, environmental barriers (Nguyen, 2020), perceived environmental factors (Dolce et al., 2018), entrepreneurial passion (Saboor et al., 2020) and entrepreneurial intention.

According to the social learning theory, this study held that entrepreneurial role models could enhance the level of entrepreneurial perceived behavioral control of college students. In the process of interacting with entrepreneurial role models, college students find common or similar points in terms of entrepreneurial ability and entrepreneurial resources through comparison with entrepreneurial role models, so as to enhance their recognition of the feasibility of entrepreneurship. In addition, college students can observe or feel the attitudes, values and behavior patterns that are positively reinforced by the alternative results of entrepreneurial role models from the behaviors, suggestions and recommendations of entrepreneurial role models, and imitate them, thus enriching entrepreneurial knowledge and experience and improving entrepreneurial ability (Bandura et al., 1999). Empirical studies showed that entrepreneurial ability was positively associated with confidence in entrepreneurial ability (Mozahem and Adlouni, 2021). At the same time, confidence in their own entrepreneurial ability as a prominent feature of entrepreneurial role models is also observed, felt and imitated by college students. In addition, entrepreneurial role models can enhance college students’ confidence in entrepreneurial resources, since they provide college students guide and help on entrepreneurship. Nguyen (2020) found that individual perceived access to finance played significant roles in the development of entrepreneurial perceived behavioral control.

To sum up, entrepreneurial role models can enhance college students’ confidence in entrepreneurial ability and resources, that is, they can enhance their entrepreneurial perceived behavioral control. Based on this, this study put forward the following hypotheses:

*H2*: Entrepreneurial role models are positively associated with college students’ entrepreneurial perceived behavioral control.

According to the theory of planned behavior, entrepreneurial perceived behavioral control is an important variable to predict entrepreneurial intention. Some empirical studies have proved that entrepreneurial perceived behavioral control is positively associated with entrepreneurial intention (Krueger et al., 2000; Sesen, 2012; Hui-Chen et al., 2014; Bux, 2016; Karimi et al., 2016; Utami, 2017). Those who have a more positive view of their abilities and resources view entrepreneurship more as an opportunity than a risk, which makes them more inclined to start a business.

Entrepreneurial desire is component of entrepreneurial calling. The research of Perugini and Bagozzi (2001) showed that entrepreneurial desire was the mediating variable of entrepreneurial perceived behavioral control and entrepreneurial intention. And they found that entrepreneurial desire was the direct determinant of entrepreneurial intention and provided the direct impetus for entrepreneurial intention. Entrepreneurial desire has lower expressivity and behavioral connectivity than entrepreneurial intention (Perugini and Bagozzi, 2004).

In addition, entrepreneurial self-efficacy is an important component of entrepreneurial perceived behavior control. According to social learning theory, confidence in an individual’s own ability positively affects the evaluation of his activities and emotional experience (Bandura, 1986). Previous studies have already found connections between confidence in person’s own ability and emotions (DeMauro and Jennings, 2016; Hascher and Hagenauer, 2016; Mahler et al., 2017). Moreover, the empirical study of Cardon and Kirk (2015) showed that individuals were more inclined to identify with the activities they were confident to engage in, so as to realized self-identity; and the stronger the individuals’ confidence in the ability towards an activity was, the stronger the desire to engage in the activity was. The empirical study of Baum and Locke (2004) showed that the expectation of success brought about by individuals’ confidence in the ability to engaged in a certain activity would lead to their strong positive emotion for the activity.

It can be concluded from the aforementioned that the improvement of entrepreneurial perceived behavioral control of college students can enhance the positive emotion about entrepreneurship, entrepreneurial identity and entrepreneurial desire, which is to say, the improvement of entrepreneurial perceived behavioral control of college students can enhance their entrepreneurial calling.

To sum up, based on hypothesis 2, the following hypothesis was proposed in this study:

*H3*: Entrepreneurial perceived behavioral control mediates the association between entrepreneurial role models and college students’ entrepreneurial calling.

### 2.3. The moderating role of entrepreneurial hands-on practice

Entrepreneurial role model demonstration and entrepreneurial hands-on practice are two important ways of entrepreneurial practice education (Zozimo et al., 2017; Hou et al., 2019; Boldureanu et al., 2020; Huang et al., 2021; Lv et al., 2021; San-Martin et al., 2021; Li et al., 2022; Luo M. et al., 2022). Entrepreneurial hands-on practice includes simulated and actual entrepreneurial hands-on practice (Piperopoulos and Dimov, 2014). Entrepreneurship role model demonstration can help college students accumulate the indirect experience in entrepreneurship, while entrepreneurial hands-on practice can enrich college students’ direct experience in entrepreneurship.

In the process of entrepreneurial role models’ influencing entrepreneurial calling of college students, this study questioned whether entrepreneurial hands-on practice may play a positive role. This study thought that compared with those who had not participated in entrepreneurial hands-on practice, college students participating in entrepreneurial hands-on practice could enhance their intuitive understanding and first-hand experience of entrepreneurial activities and entrepreneurial environment, and improved their entrepreneurial knowledge, skills and ability. Furthermore, entrepreneurial perceived behavioral control significantly enhances entrepreneurial ability (Seikkula-Leino and Salomaa, 2020), and entrepreneurial ability significantly enhances entrepreneurial perceived behavioral control (Mozahem and Adlouni, 2021). Entrepreneurial perceived behavioral control and entrepreneurial ability can promote each other. To sum up, based on hypothesis 3, the following hypothesis was proposed in this study:

*H4*: College students’ entrepreneurial hands-on practice positively moderates the link between entrepreneurial perceived behavioral control and entrepreneurial calling.

On the basis of the above discussion, this study held that entrepreneurial role models were indirectly associated with entrepreneurial calling through the mediation of entrepreneurial perceived behavioral control, while entrepreneurial hands-on practice played a moderating role in this process. That was, it was a moderated mediation model. Entrepreneurial perceived behavioral control mediated the association between entrepreneurial role models and entrepreneurial calling, and the strength of the mediating role was moderated by entrepreneurial hands-on practice. Based on this, the following hypothesis was proposed in this study:

*H5*: College students’ entrepreneurial hands-on practice moderates the mediating role of entrepreneurial perceived behavioral control between entrepreneurial role models and entrepreneurial calling.

### 2.4. The present study: A moderated mediation model

**Figure 1:**
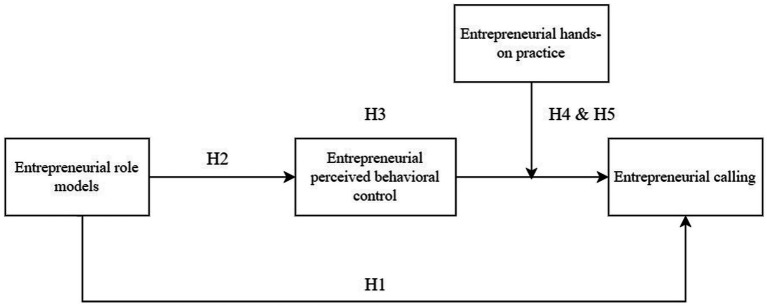
The mediated moderation model.

This study established the moderated mediation model to test these hypotheses (Figure 1). Firstly, the positive association of entrepreneurial role models with entrepreneurial calling was tested (Hypothesis 1). Secondly, the positive association of entrepreneurial role models with college students’ entrepreneurial perceived behavioral control was examined (Hypothesis 2). Thirdly, the mediating role of entrepreneurial perceived behavioral control in the association of entrepreneurial role models with college students’ entrepreneurial calling was tested (Hypothesis 3). Fourthly, the positively moderating role of college students’ entrepreneurial hands-on practice in the association of entrepreneurial perceived behavioral control with entrepreneurial calling was examined (Hypothesis 4). In the end, the moderating role of entrepreneurial hands-on practice in the mediating role of entrepreneurial perceived behavioral control between entrepreneurial role models and entrepreneurial calling was tested (Hypothesis 5).

## 3. Method

### 3.1. Participants and procedure

This study was a survey. A preliminary survey was conducted among 20 college students in Beijing Technology and Business University 1 month before the formal survey, and the questionnaires were revised accordingly. Participants in this study were students of colleges and universities from China. A convenience sampling technique was used. The 2018 China Mass Entrepreneurship Index Report released by the Innovation and Entrepreneurship Research Center of Southwest Jiaotong University ranked China’s actual performance in innovation and entrepreneurship by region (Guo et al., 2022). To ensure the validity and generality of our results, we took into account the entrepreneurial regions of the surveyed colleges and universities when collecting data. Five surveyed universities (Tsinghua university, Renmin University of China, Beijing Technology and Business University, Shandong Science and Technology University and Ludong University) came from the top five entrepreneurial regions, including Beijing and Shandong; 10 surveyed universities (Shanghai Finance and Economics University, Shanghai Lixin Accounting and Finance University, Henan University, Hubei University, Geological University of China, Sichuan University, Tianjin University, Nankai University, Hebei University and Langfang Normal University) came from mid-ranking entrepreneurial regions, including Shanghai, Henan, Hubei, Sichuan, Tianjin and Hebei; and 1 surveyed college (Xinjiang Institute of Engineering) came from the bottom five entrepreneurial regions, including Xinjiang.

The investigation process is that firstly some teachers from aforementioned colleges and universities were communicated with, and then they organized college students to fill in the paper questionnaires during the break time after class. The paper questionnaires were divided into three parts. The first part includes respondent basic information questionnaire, entrepreneurial role models scale and entrepreneurial hands-on practice questionnaire. The second part is entrepreneurial perceived behavioral control scale. The third part is entrepreneurial calling scale. The questionnaires were coded to ensure that the respondents of the three parts are consistent. The respondents answered these three parts of the questionnaires every 3 months. 627 questionnaires were actually collected, and 519 valid questionnaires were obtained after removing invalid ones, with effective questionnaire recovery rate of 82.8%.

To decrease common method variance, a longitudinal survey was conducted. The survey process started with contacting 23 teachers from the above colleges and universities and asking them to invite students in the classes where they were the head teachers to participate in the survey. The paper questionnaires were divided into three parts. The first part includes respondent basic information questionnaire, entrepreneurial role models scale and entrepreneurial hands-on practice questionnaire. The second part is entrepreneurial perceived behavioral control scale. The third part is entrepreneurial calling scale. The questionnaires were coded to ensure the respondents of the three parts are consistent. The respondents answered these three parts of the questionnaires every 3 months and the study data was gathered at three points in time. In the process of data collection, the participants were promised that information related to themselves would be treated as confidential strictly. A total of 652 questionnaires were distributed and 627 questionnaires were actually collected. Eventually, 519 valid questionnaires were obtained after removing invalid ones, with valid questionnaire recovery rate of 79.6% compared to the number of distributed questionnaires and 82.8% compared to the number of collected questionnaires.

### 3.2. Sample characteristics

Among the 519 college students, 44.5 percent were male and 55.5 percent were female; 24.7 percent were freshmen, 21.4 percent were sophomore, 24.9 percent were junior, and 29.1 percent were senior (including senior five). In terms of native place, 54.9 percent of them came from urban areas and 45.1 percent from rural areas. In terms of majors, 44.1 percent majored in science and technology, 32.6 percent in economics and management, and 23.3 percent in other majors. In terms of college type, 27.7 percent of the respondents studied in national high-level universities, 26.6 percent in provincial key construction colleges and universities, and 45.7 percent in provincial general colleges and universities. In terms of region of college, 46.5 percent of them attended colleges and universities from the top five entrepreneurial regions of China, 22.5 percent attended colleges and universities from mid-ranking entrepreneurial regions and 31.0 percent attended colleges and universities from the bottom five entrepreneurial regions.

### 3.3. Measures

The scales of entrepreneurial role models, entrepreneurial perceived behavioral control and entrepreneurial calling, adopted in the present study, are mature scales. To ensure the accuracy of the translation, we used the back translation method (Breslin, 1970). Two postgraduates in the direction of the organizational behavior research translated the original English version of the scales into Chinese, then two other postgraduates in the same research direction back-translated the Chinese version of the scales into English, and then compared the original English version scales and the back-translated English version scales. After finding the difference between the two version scales, adjusted the Chinese version scales accordingly. The Chinese version scales were used in this questionnaire survey. All the scales were rated using 7-point Likert-type scale ranging from 1 (strongly disagree) to 7 (strongly agree). In addition, this study originally developed the entrepreneurial hands-on practice questionnaire and the respondent basic information questionnaire. The details are as follows.

#### 3.3.1. Entrepreneurial role models

The variable “Entrepreneurial role models” was measured using the 5-item scale developed by Fellnhofer and Mueller (2018). Sample items included: “There is an entrepreneurial person I am trying to be like in my career pursuits” and “There is an entrepreneurial person particularly inspirational to me in my career path.” In this study, Cronbach’s Alpha value of this scale was 0.88.

#### 3.3.2. Entrepreneurial perceived behavioral control

Entrepreneurial perceived behavioral control was measured using the 6-item scale developed by Liñán and Chen (2009). Sample items included: “To start a firm and keep it working would be easy for me” and “I know the necessary practical details to start a firm.” In this study, Cronbach’s Alpha value of this scale was 0.92.

#### 3.3.3. Entrepreneurial calling

Entrepreneurial calling was measured using the 12-item scale developed by Dobrow and Tosti-Kharas (2011). Sample items included: ‘I am passionate about entrepreneurship’ ‘I enjoy being an entrepreneur more than anything else’ and ‘Being an entrepreneur gives me immense personal satisfaction.’ In this study, Cronbach’s Alpha value of this scale was 0.93.

#### 3.3.4. Entrepreneurial hands-on practice

The entrepreneurial hands-on practice questionnaire was originally developed. The measured variables are binary variables, with “1” indicating having participated in the entrepreneurial practice personally (including entrepreneurial simulation competition, entrepreneurial simulation training, entrepreneurial park incubation project, venture fund support project, spontaneous entrepreneurship, etc.), and “0” indicating having not participated in the entrepreneurial practice personally.

#### 3.3.5. Control variables

The possible control variables were investigated by the self-developed respondent basic information questionnaire. Since the respondents’ entrepreneurial perceived behavioral control was significantly negatively correlated with gender (*r* = −0.12, *p* < 0.01), major (*r* = −0.11, *p* < 0.01), and significantly positively correlated with native place (*r* = 0.11, *p* < 0.01), college type (*r* = 0.17, *p* < 0.01) and region of college (*r* = 0.26, *p* < 0.01); entrepreneurial calling was significantly negatively correlated with grade (*r* = −0.10, *p* < 0.05), and significantly positively correlated with college type (*r* = 0.12, *p* < 0.01) and region of college (*r* = 0.12, *p* < 0.01); entrepreneurial hands-on practice was significantly positively correlated with grade (*r* = 0.13, *p* < 0.01; see Table 1), in the subsequent statistical analysis process to confirm the theoretical model, the control variables, including gender, major, native place, college type, region of college and grade, were added to eliminate their influence on statistics results.

**Table 1:** Descriptive statistics.

Variables	Mean	SD	1	2	3	4	5	6	7	8	9
1. Gender	1.55	0.50									
2. Grade	2.61	1.19	0.01								
3. Major	1.94	1.06	0.04	0.10^*^							
4. Native place	1.45	0.50	0.04	0.05	−0.18^**^						
5. College type	2.98	1.16	0.04	−0.20^**^	−0.09^*^	0.18^**^					
6. Region of college	1.85	0.87	0.02	−0.35^**^	−0.24^**^	0.19^**^	0.65^**^				
7. ERM	4.24	1.02	0.00	0.00	0.00	0.04	0.04	0.04			
8. EPBC	3.58	1.01	−0.12^**^	0.01	−0.11**	0.11^**^	0.17^**^	0.26^**^	0.30^**^		
9. EC	4.21	0.92	−0.03	−0.10^*^	0.02	0.04	0.12^**^	0.12^**^	0.30^**^	0.24^**^	
10. EHP	0.17	0.37	−0.06	0.13^**^	−0.01	0.03	0.04	−0.03	0.14^**^	0.23^**^	0.18^**^

*N* = 519; ERM, entrepreneurial role models; EPBC, entrepreneurial perceived behavioral control; EC, entrepreneurial calling; EHP, entrepreneurial hands-on practice. *p < 0.05; **p < 0.01.

In this study, SPSS 26.0 and AMOS 22.0 were mainly used for statistical analysis.

## 4. Results

### 4.1. Common method bias test and multi-collinearity test

The data in this study were all self-reported. Although the questionnaires were filled in anonymously, and the data was promised to be kept confidential and only used for scientific research, there still might be common method deviation of the data. Therefore, the single-common-method-factor approach was adopted to test the common method deviation of the data. First, a confirmatory factor analysis model (see Three-factor model in Table 2) was constructed. Then, on the basis of the confirmatory factor analysis model, a method latent variable was added so that all the measurement items were loaded not only on the corresponding construct factor, but also on the method latent variable. The main fitting index of the new model was as follows: χ^2^/df = 3.398, CFI = 0.937, TLI = 0.921, IFI = 0.937, NFI = 0.913, RMSEA = 0.068, SRMR = 0.0434. Finally, the main fitting indexes of the two models were compared as follows: △CFI = 0.005, △TLI = 0.001, △IFI = 0.005, △NFI = 0.007, △RMSEA = 0.001, △SRMR = 0.017. It can be seen that the changes of these fitting indices were all less than 0.03, indicating that the original model was not significantly improved after the common method deviation latent variable was added, thus indicating that there was no serious common method deviation in the data. In addition, the tolerance of entrepreneurial role models, entrepreneurial perceived behavioral control, and entrepreneurial calling is between 0.472 and 0.997. Correspondingly, the variance inflation factor (VIF) was between 1.003 and 2.119, which was lower than the critical value of 2.5. Therefore, the degree of multi-collinearity among the variables in this study was relatively low.

**Table 2:** Confirmatory factor analysis.

Models	χ^2^/df	CFI	TLI	IFI	NFI	RMSEA	SRMR
Three-factor model (ERM, EPBC, EC)	3.349	0.932	0.922	0.932	0.906	0.067	0.0604
Two-factor model (ERM + EPBC, EC)	8.043	0.791	0.767	0.792	0.770	0.117	0.1103
Single-factor model (ERM + EPBC+EC)	12.976	0.647	0.603	0.648	0.630	0.152	0.1640

ERM, entrepreneurial role models; EPBC, entrepreneurial perceived behavioral control; EC, entrepreneurial calling.

### 4.2. Reliability and validity test

As shown in Table 3, this study employed the Cronbach’s Alpha values and composite reliability (CR) to test the reliability. In this study, Cronbach’s Alpha values of the entrepreneurial role models scale, the entrepreneurial perceived behavioral control scale and the entrepreneurial calling scale were 0.88, 0.92, and 0.93, which all are greater than 0.80; the coefficients of CR range from 0.88–0.93, which all are greater than 0.70. The above data showed that these three scales have good homogeneity reliability.

**Table 3:** Reliability and validity tests.

Constructs	Items	Factor loading	Cronbach’s α	CR	AVE
**Entrepreneurial role models**	There is an entrepreneurial person I am trying to be like in my career pursuits	0.767	0.88	0.88	0.60
	There is an entrepreneurial person particularly inspirational to me in my career path	0.865			
	In the career path I am pursuing, there is an entrepreneurial person I admire	0.822			
	I have a mentor in my potential entrepreneurial career field	0.694			
	I know of an entrepreneurial person who has a career I would like to pursue	0.710			
**Entrepreneurial perceived behavioral control**	To start a firm and keep it working would be easy for me	0.758	0.92	0.92	0.65
	I know the necessary practical details to start a firm	0.773			
	I know how to develop an entrepreneurial project	0.849			
	I can control the creation process of a new firm	0.782			
	If I tried to start a firm, I would have a high probability of succeeding	0.871			
	I am prepared to start a viable firm	0.779			
**Entrepreneurial calling**	I am passionate about entrepreneurship	0.749	0.93	0.93	0.51
	I enjoy being an entrepreneur more than anything else	0.763			
	Being an entrepreneur gives me immense personal satisfaction	0.688			
	I would sacrifice everything to be an entrepreneur.	0.652			
	The first thing I often think about when I describe myself to others is that I’m an entrepreneur	0.680			
	I would continue being an entrepreneur even in the face of severe obstacles	0.755			
	I know that being an entrepreneur will always be part of my life	0.706			
	I feel a sense of destiny about being an entrepreneur	0.679			
	Being an entrepreneur is always in my mind in some way	0.732			
	Even when not starting a business, I often think about being an entrepreneur	0.748			
	My existence would be much less meaningful without my being an entrepreneur	0.693			
	Being an entrepreneur is a deeply moving and gratifying experience for me	0.705			

The average variance extraction values (AVE) of entrepreneurial role models, entrepreneurial perceived behavioral control and entrepreneurial calling were calculated by maximum likelihood estimation method, and were 0.60, 0.65, and 0.51 respectively, which all were greater than 0.5, indicating that these three scales had good convergent validity.

Confirmatory factor analysis was used to test the discriminant validity of the entrepreneurial role models scale, the entrepreneurial perceived behavioral control scale and the entrepreneurial calling scale. First, three models were constructed, which were three-factor model constructed by entrepreneurial role models, entrepreneurial perceived behavioral control and entrepreneurial calling, two-factor model combining entrepreneurial role models and entrepreneurial perceived behavioral control, and one-factor model combining the above three variables. Then confirmatory factor analysis was carried out for the three models. Finally compared the fitting indexes of these three models. The results showed that all the three-factor model’s indicators were dominant, and the model’s fitting index was the best (χ^2^/df = 3.349 < 5, CFI = 0.932 > 0.9, TLI = 0.922 > 0.9, IFI = 0.932 > 0.9, NFI = 0.906 > 0.9, RMSEA = 0.067 < 0.08, SRMR = 0.0604 < 0.08, see Table 2), indicating that these three scales had good discriminant validity.

### 4.3. Multivariate normal test

**Figure 2:**
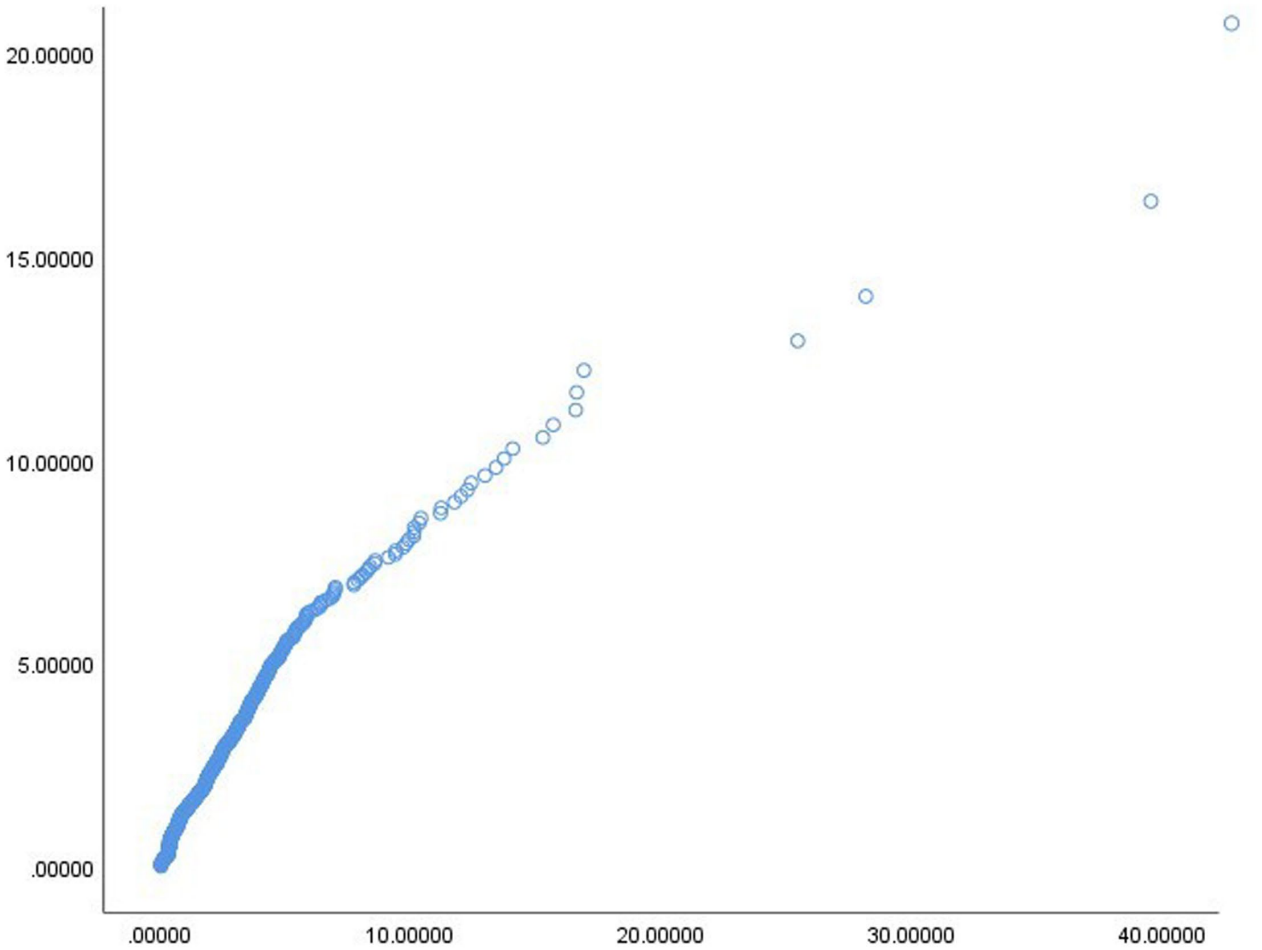
Chi-square and Mahalanobis distance plot.

Kim (2013) stated that if the |skewness| > 2 or |kurtosis| > 7, the data did not form a normal distribution. Table 4 showed the skewness and kurtosis of each variable. The results showed that the sample presented a normal distribution. Additionally, if the variables form a multivariate normal distribution, the Chi-square and Mahalanobis distance plot will form a straight line (Nor, 2015). Figure 2 showed that the variables of this sample formed a multivariate normal distribution.

**Table 4:** Skewness and kurtosis of each variable.

Variables	Skewness	Kurtosis
Entrepreneurial role models	−0.382	0.965
Entrepreneurial perceived behavioral control	−0.255	−0.169
Entrepreneurial calling	−0.367	0.141

### 4.4. Descriptive statistics analysis

As shown in Table 1, the mean of entrepreneurial role models, entrepreneurial perceived behavioral control and entrepreneurial calling of college students were between 3.5 and 4.5, meaning medium level. The proportion of college students who had participated in entrepreneurial hands-on practice was relatively small, accounting for 17%. The above data showed that there was great space to improve for college students in entrepreneurship role models, entrepreneurial perceived behavioral control, entrepreneurial calling and entrepreneurial hands-on practice.

Table 1 also showed that entrepreneurial role models were positively correlated with entrepreneurial perceived behavioral control (*r* = 0.30, *p* < 0.01), entrepreneurial calling (*r* = 0.30, *p* < 0.01) and entrepreneurial hands-on practice (*r* = 0.14, *p* < 0.01). Entrepreneurial perceived behavioral control was positively correlated with entrepreneurial calling (*r* = 0.24, *p* < 0.01) and entrepreneurial hands-on practice (*r* = 0.23, *p* < 0.01). Entrepreneurial calling was positively correlated with entrepreneurial hands-on practice (*r* = 0.18, *p* < 0.01). The significant correlation between these four variables provided preliminary support for the verification of the theoretical model, and showed that these variables could be further statistically analyzed.

### 4.5. Main role test

Hierarchical regression analysis was used to test the hypothesis of the direct association of entrepreneurial role models with entrepreneurial calling. Model 2 in Table 5 showed that entrepreneurial role models were positively associated with college students’ entrepreneurial calling (β = 0.296, *p* < 0.001), and hypothesis H1 has been verified.

**Table 5:** Hierarchical linear regression analysis results.

Variables	EC					EPBC	
	Model 1	Model 2	Model 3	Model 4	Model 5	Model 6	Model 7
**Controls**							
Gender	−0.040	−0.039	−0.021	−0.007	−0.003	−0.123^**^	−0.122^**^
Grade	−0.072	−0.075	−0.090	−0.111^*^	−0.112^*^	0.105^*^	0.102^*^
Major	0.052	0.049	0.056	0.064	0.061	−0.041	−0.045
Native place	0.031	0.021	0.014	0.018	0.017	0.055	0.045
College type	0.061	0.055	0.057	0.047	0.057	−0.004	−0.009
Region of college	0.067	0.060	0.020	0.021	0.006	0.280^***^	0.273^***^
**Independent**							
ERM		0.296^***^	0.253^***^				0.292^***^
**Mediator**							
EPBC			0.147^**^	0.195^***^	0.207^***^		
**Moderator**							
EHP				0.145^**^	0.086		
**Interaction**							
EPBC×EHP					0.137^**^		
R^2^	0.026	0.113	0.131	0.093	0.108	0.97	0.182
R^2^ change	0.026	0.087	0.018	0.067	0.015	0.97	0.085
F	2.301^*^	9.345^***^	9.624^***^	6.529^***^	6.868^***^	9.178^***^	16.257^***^

*N* = 519; ERM, entrepreneurial role models; EPBC, entrepreneurial perceived behavioral control; EC, entrepreneurial calling; EHP, entrepreneurial hands-on practice. *p < 0.05; **p < 0.01; ***p < 0.001.

### 4.6. Mediating role test

Hierarchical regression analysis was used to test the hypothesis of the mediating role of entrepreneurial perceived behavioral control between entrepreneurial role models and entrepreneurial calling. Model 7 in Table 5 showed that entrepreneurial role models were significantly positively associated with entrepreneurial perceived behavioral control (β = 0.292, *p* < 0.001), and hypothesis H2 was verified. Model 2 and Model 3 in Table 5 showed that after incorporating entrepreneurial perceived behavioral control into the regression equation, the coefficient of association of entrepreneurial role models with entrepreneurial calling had dropped from 0.296 (*p* < 0.001) to 0.253 (*p* < 0.001), indicating that the association of entrepreneurial role models with entrepreneurial calling weakened. At the same time, entrepreneurial perceived behavioral control was significantly positively associated with entrepreneurial calling (β = 0.147, *p* < 0.01), indicating that entrepreneurial perceived behavioral control played a partially mediating role in the association of entrepreneurial role models with college students’ entrepreneurial calling, and hypothesis H3 has been verified.

### 4.7. Moderation role tests

Hierarchical regression analysis was used to test the moderating role of entrepreneurial hands-on practice between entrepreneurial perceived behavioral control and entrepreneurial calling. Firstly, the standardized entrepreneurial perceived behavioral control and entrepreneurial hands-on practice were multiplied to construct an interactive term. Then, taking entrepreneurial calling as the dependent variable, gradually introduce control variables, entrepreneurial perceived behavioral control, entrepreneurial hands-on practice, and interactive item of entrepreneurial perceived behavioral control and entrepreneurial hands-on practice. Model 5 in Table 5 showed that the interactive term of entrepreneurial perceived behavioral control and entrepreneurial hands-on practice were significantly positively associated with entrepreneurial calling (β = 0.137, *p* < 0.01), which means when college students had entrepreneurial hands-on practice, entrepreneurial perceived behavioral control were more strongly positively associated with entrepreneurial calling, and hypothesis H4 has been verified.

**Figure 3:**
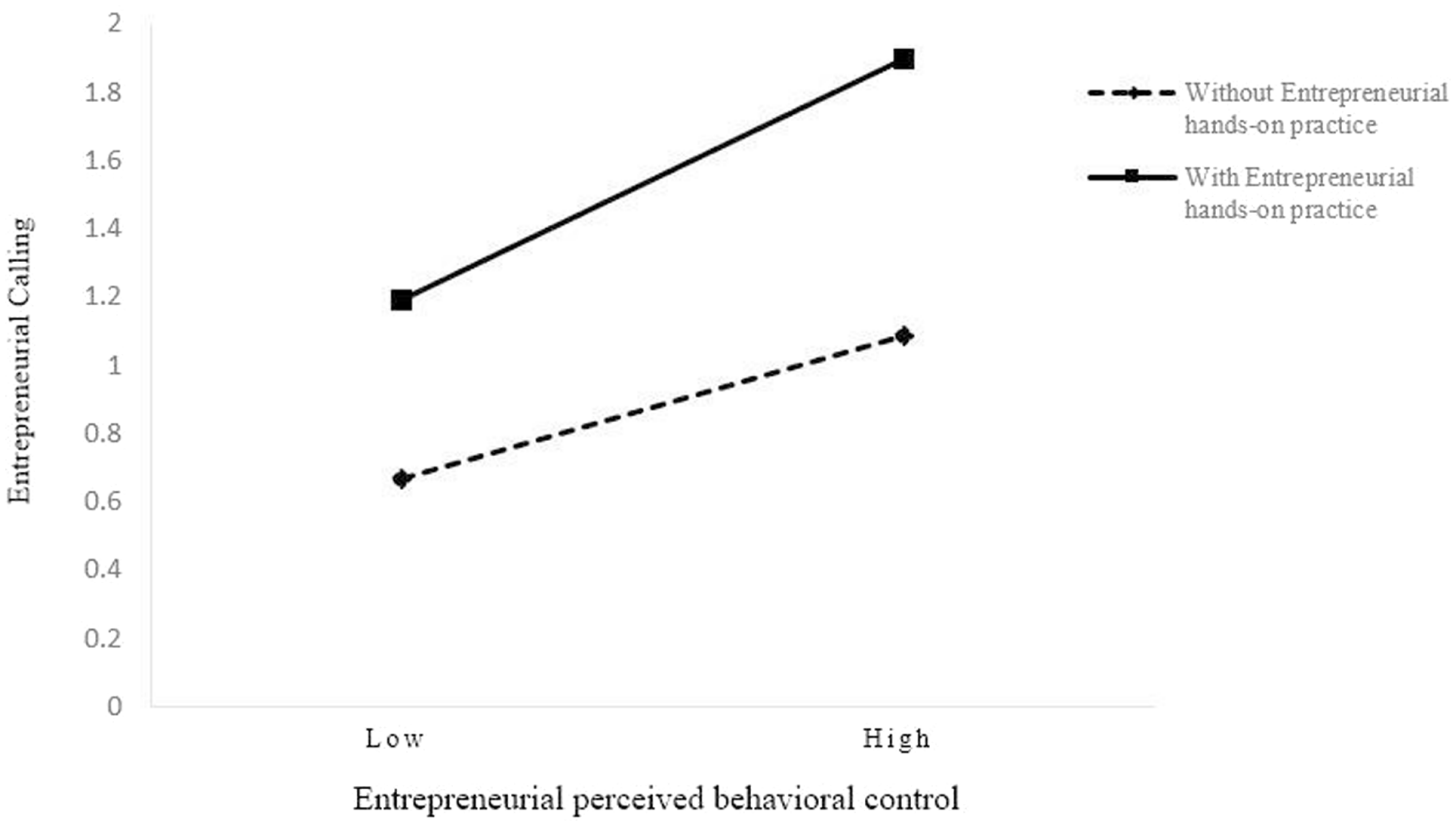
Entrepreneurial hands-on practice as a moderator between entrepreneurial perceived behavioral control and entrepreneurial calling.

In order to further clarify the moderating role of entrepreneurial hands-on practice, according to the suggestion of Cohen et al. (2003), this study plotted simple slopes to show the relationship between entrepreneurial perceived behavioral control and entrepreneurial calling with and without entrepreneurial hands-on practice, as shown in Figure 3. The simple slope test result showed that the slope of the line with entrepreneurial hands-on practice (γ = 0.471, *p* < 0.001) was larger than the slope of the line without entrepreneurial hands-on practice (γ = 0.133, *p* < 0.01), indicating that perceived behavioral control was significantly positively associated with entrepreneurial calling regardless of entrepreneurial hands-on practice, and under the condition of entrepreneurial hands-on practice, entrepreneurial perceived behavioral control was significantly positively associated with entrepreneurial calling.

### 4.8. Moderated mediation role test

In this study, the PROCESS in SPSS developed by Hayes (2013) was used to test the moderating role of entrepreneurial hands-on practice on the mediating role of entrepreneurial perceived behavioral control. The results are shown in Table 6. When respondents had entrepreneurial hands-on practice, the indirect association of entrepreneurial role models with entrepreneurial calling through entrepreneurial perceived behavioral control was significant (b = 0.1193, Boot 95% CI does not include 0). When respondents had no entrepreneurial hands-on practice, the indirect association of entrepreneurial role models with entrepreneurial calling through entrepreneurial perceived behavioral control was not significant (b = 0.0180, Boot 95% CI includes 0), indicating that entrepreneurial hands-on practice moderated the mediating role of entrepreneurial perceived behavioral control between entrepreneurial role models and entrepreneurial calling, and hypothesis H5 has been verified.

**Table 6:** Results of moderated mediation model test.

Moderator	ERM→EPBC→EC		
	Indirect role	SE	95%CI
With entrepreneurial hands-on educational practice	0.1193	0.0281	[0.0686, 0.1789]
Without entrepreneurial hands-on educational practice	0.0180	0.0163	[−0.0133, 0.0516]

*N* = 519; ERM, entrepreneurial role models; EPBC, entrepreneurial perceived behavioral control; EC, entrepreneurial calling.

**Figure 4:**
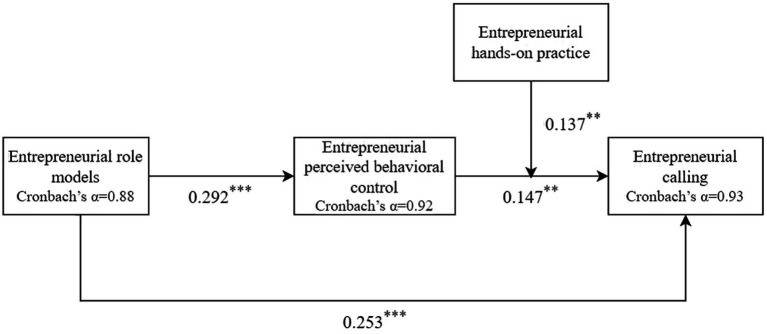
A moderated mediation model of the association between entrepreneurial role models and entrepreneurial calling through entrepreneurial perceived behavioral control, entrepreneurial hands-on practice. Cronbach’s Alpha values and path coefficients are shown. ^**^p < 0.01; ^***^p < 0.001.

Figure 4 showed the path coefficients in the moderated mediation model and the Cronbach’s Alpha values of the variable scales.

## 5. Discussion

The current study aims to assess the association between entrepreneurial role models and entrepreneurial calling of college students through the mediating role of entrepreneurial perceived behavioral control and the moderating role of entrepreneurial hands-on practice based on the social learning theory, the theory of planned behavior and the entrepreneurial event model. All of the study hypotheses were confirmed.

H1 contends that entrepreneurial role models have significant positive association with entrepreneurial calling. That means as the exemplary role of entrepreneurial role models increases, college students’ entrepreneurial calling is enhanced. That is because passion for entrepreneurship, recognition of entrepreneurial value and entrepreneur identity are the defining characteristics of entrepreneurial role models (Zhang and Chun, 2018; Saboor et al., 2020; Wang and Jiang, 2021; Zhang et al., 2021). In the process of following and imitating entrepreneurial role models, college students will observe and learn these characteristics of entrepreneurial role models, develop entrepreneurial desire, and then their entrepreneurial calling will be strengthened. The previous studies have showed that entrepreneurial role models have significant positive association with college students’ aspirations of entrepreneurship (Meccheri and Pelloni, 2006). Entrepreneurial desire is the antecedent of an individual’s entrepreneurial intention (Shapero and Sokol, 1982; Peterman and Kennedy, 2003). Entrepreneurial role models have significant positive association with entrepreneurial intention (Fellnhofer, 2017b; Fellnhofer and Mueller, 2018; Van Trang et al., 2019; San-Martin et al., 2021). Therefore, the previous studies are aligned with the results of the current study. Thus, it can be seen that the more college students follow and emulate entrepreneurial role models, the stronger their entrepreneurial calling is.

H2 and H3 contend that entrepreneurial perceived behavioral control partially mediates the relationship between entrepreneurial role models and entrepreneurial calling. The partially mediating role manifests that as the exemplary role of entrepreneurial role models increases, college students’ entrepreneurial perceived behavioral control intensifies, which then facilitates their entrepreneurial calling. That’s because in the process of following and emulating entrepreneurial role models, entrepreneurial role models can play exemplary roles and provide encouragement, guidance and help on entrepreneurship for college students, so college students will increase confidence in the ability and resources about entrepreneurship. And since entrepreneurship can meet college students’ demands including autonomy, competence and social connection (Al-Jubari et al., 2018), college students’ entrepreneurial calling will be enhanced. The previous studies have showed that entrepreneurial role models have significant positive association with entrepreneurial perceived behavioral control (Fellnhofer, 2017b; Fellnhofer and Mueller, 2018; Van Trang et al., 2019; San-Martin et al., 2021), and its similar variables, including entrepreneurial competence (Van Trang et al., 2019), and entrepreneurial perceived feasibility (Zozimo et al., 2017). Entrepreneurial perceived behavioral control is positively associated with entrepreneurial intention (Chien-Chi et al., 2020; Huang and Zhang, 2020). Entrepreneurial desire was the mediating variable of entrepreneurial perceived behavioral control and entrepreneurial intention (Perugini and Bagozzi, 2001). Therefore, the past studies are aligned with the results of this study.

H4 and H5 contend that entrepreneurial hands-on practice moderates not only the positive association of entrepreneurial perceived behavioral control and entrepreneurial calling, but also the mediating role of entrepreneurial perceived behavioral control between entrepreneurial role models and entrepreneurial calling. The moderating role manifests that for college students with personal entrepreneurial practice, the improvement of their entrepreneurial perceived behavioral control can bring more enhancement of entrepreneurial calling. In addition, entrepreneurial role models can enhance their more entrepreneurial calling by improving their entrepreneurial perceived behavioral control. That’s because college students who participate in hands-on entrepreneurship practice can enhance entrepreneurial ability based on intuitive understanding and personal experience on entrepreneurial activities and entrepreneurial environment. This part of entrepreneurial ability cannot be replaced by that part of entrepreneurial ability based on indirect experience learned from entrepreneurial role models. Entrepreneurial perceived behavioral control and this part of entrepreneurial ability promote each other, thus enhancing entrepreneurial calling. Calling is influenced by behavioral participation (Winner, 2000). The combination of college students’ direct experience from their own entrepreneurial experience and indirect experience from entrepreneurial role models can further promote entrepreneurial calling. The previous studies have showed entrepreneurial practice is positively associated with college students’ entrepreneurial intention (Huang et al., 2021; Wang J. et al., 2022). In the association between entrepreneurial theory education and entrepreneurial intention through entrepreneurial self-efficacy, entrepreneurial practice plays the moderating role (Wang J. et al., 2022). Entrepreneurship competitions are positively associated with college students’ entrepreneurial intention (Huang et al., 2021; Lv et al., 2021; Li et al., 2022; Wang J. et al., 2022). In the current study, entrepreneurship competitions belong to entrepreneurial hands-on practice. Therefore, the past studies are aligned with the results of this study.

### 5.1. Theoretical implications

This study has the following theoretical implications.

First, this research studies the association between entrepreneurial role models and entrepreneurial calling based on the social learning theory. The results of this study support the discussion about entrepreneurial role models and entrepreneurial calling, and expand the application of the social learning theory. The study on the effects of entrepreneurial role models is a major theme of entrepreneurship education research. Previous studies have studied the influence of entrepreneurial role models on entrepreneurial intention, entrepreneurial behavior control, entrepreneurial competence, entrepreneurial feasibility, and entrepreneurial failure fear (Van Trang et al., 2019). This study advances the field by examining entrepreneurial calling - the important but neglected outcome variable. In our research, we view entrepreneurship as a vocation, different from previous studies, treating entrepreneurship as a general activity. Since calling is significantly positively correlated with occupational commitment (Sawhney et al., 2020; Jia et al., 2021; Direnzo et al., 2022) and career choice (Hirschi et al., 2018), that means cultivating college students’ entrepreneurial calling is valuable in entrepreneurship education. However, there is almost no research on entrepreneurial calling in entrepreneurship education so far. Considering that entrepreneurs have strong entrepreneurial calling (Tian et al., 2018), and entrepreneurial role model is an important factor affecting college students’ career exploration (Luo M. et al., 2022), we put forward and verify entrepreneurial role models are positively associated with college students’ entrepreneurial calling. This research also promotes the research of entrepreneurial calling.

Second, this research studies the mediating role of entrepreneurial perceived behavioral control based on the social learning theory, the theory of planned behavior and the entrepreneurial event model. The findings of this study add to the understanding of planned behavior theory and the entrepreneurial event model, and relate them to the social learning perspective of entrepreneurship, thus contributing to the theoretical integration of entrepreneurship education. This study confirms the concept of entrepreneurial perceived behavioral control covers that concept of recognition of entrepreneurship feasibility, entrepreneurial perceived behavioral control is an antecedent of entrepreneurial desire, and they all can be learned from entrepreneurial role models. Entrepreneurial role models are the objects that college students can follow on profession, but how they influence college students’ career development and career choice? This study supports college students can enhance their entrepreneurial calling through improving entrepreneurial perceived behavioral control in the process of following and emulating entrepreneurial role models. This study also expands the literature on how to enhance college students’ entrepreneurial calling.

Third, the study reveals entrepreneurial hands-on practice moderates the association between entrepreneurial role models and entrepreneurial calling, expanded the literature on the relationship among entrepreneurial hands-on practice, entrepreneurial role models, entrepreneurial perceived behavioral control and entrepreneurial calling. For college students with hands-on practice of entrepreneurship, entrepreneurial role models have more influence on entrepreneurial calling through entrepreneurial perceived behavioral control. Entrepreneurial role models mainly improve college students’ indirect experience in entrepreneurship, while entrepreneurial hands-on practice mainly improves college students’ direct experience in entrepreneurship. The two mode of entrepreneurship practice education cannot replace each other, and instead need to be integrated. The results of this study suggest that sufficient research on entrepreneurial hands-on practice in the field of entrepreneurship education is necessary (Wu et al., 2022). In the past, the research in this area focused more on the entrepreneurial competitions, and the scope of entrepreneurial hands-on practice can be expanded in the future. The importance of increasing college students’ intuitive understanding and personal experience about entrepreneurship in entrepreneurship education can also be discussed in more depth.

### 5.2. Practical implications

The results of this study provide some useful and practical suggestions.

First, since entrepreneurial role models and college students’ entrepreneurial calling are positively associated, colleges and universities are suggested to increase entrepreneurial role models so as to enhance college students’ entrepreneurial calling. Specifically, colleges and universities can organize students to watch the documentary about the struggle of entrepreneurs, hire outstanding entrepreneurs as guest lecturers and business mentors. In addition, by building entrepreneurial culture and advocating entrepreneurial values, college students can be encouraged to spontaneously pay attention to entrepreneurs and actively seek out entrepreneurial role models.

Second, since entrepreneurial perceived behavioral control partially mediates the relationship between entrepreneurial role models and entrepreneurial calling, the increase of college students’ entrepreneurial perceived behavioral control should be valued. In view of this, colleges and universities are advised to introduce measurement tools of college students’ entrepreneurial perceived behavioral control, and periodically monitor its level. At the same time, various measures should be taken to enhance the level of college students’ entrepreneurial perceived behavioral control. Specifically, colleges and universities can provide entrepreneurial resources for college students, such as establishing entrepreneurial education practice bases, providing entrepreneurial platforms, raising entrepreneurial funds, and so on. They also can exercise their students’ entrepreneurial ability through opening entrepreneurship courses (Zhong et al., 2020), introducing and setting entrepreneurship models, setting up entrepreneurship park incubation projects, promoting their students to participate in entrepreneurship simulation training and competitions and so on.

Third, since college students’ entrepreneurial hands-on practice strengthened not only the link between entrepreneurial perceived behavioral control and entrepreneurial calling, but also the indirect association between entrepreneurial role models and entrepreneurial calling through entrepreneurial perceived behavioral control, colleges and universities are advised to actively expand the students’ entrepreneurial hands-on practice while they are trying to enhance the students’ entrepreneurial role models and entrepreneurial perceived behavioral control so as to promote entrepreneurial calling. Specifically, colleges and universities can provide or create more simulated and real entrepreneurship situations, and encourage the students to start their own business to promote their entrepreneurial hands-on activities.

### 5.3. Limitations and future directions

This study has several limitations that deserve scholarly attention in the future.

First, our study adopted convenience sampling method to collect data. Future research should use random sampling method to ensure representative sample so as to make generalizations more cautiously.

Second, in our study, the variables were measured from the same sources, which can bring about common method bias (Podsakoff et al., 2003). Although we employed longitudinal research method to try to mitigate the common method variance, the variance remains. To overcome this limitation, entrepreneurial calling can be measured from different sources, such as college students’ teachers or companions in future research.

Third, since there are differences between men and women in their attitudes, resources, knowledge, skills and other backgrounds about entrepreneurship (Dawson and Henley, 2015), male and female college students may respond differently to entrepreneurship education. Some studies have indicated there are gender differences in the relationship between entrepreneurial role models and entrepreneurial intention (Entrialgo and Iglesias, 2018; Torres-Coronas and Vidal-Blasco, 2018). Future research needs to explore the gender differences in the relationship between entrepreneurial role models and entrepreneurial calling.

## 6. Conclusion

This study proposed a moderated mediation model to unveil the relationship between entrepreneurial role models and entrepreneurial calling of college students, and examined the mediating role of entrepreneurial perceived behavioral control and the moderating role of entrepreneurial hands-on practice in the model. The results showed a direct and positive association between entrepreneurial role models and entrepreneurial calling. Furthermore, the partially mediating role of entrepreneurial perceived behavioral control manifested that as entrepreneurial role models increased, entrepreneurial perceived behavioral control intensified, which then facilitated entrepreneurial calling. Moreover, the moderating role of entrepreneurial hands-on practice showed with entrepreneurial hands-on practice, not only the positive association of entrepreneurial perceived behavioral control and entrepreneurial calling, but also the mediating role of entrepreneurial perceived behavioral control is stronger. This study innovatively discussed the mechanism and boundary condition of the association between entrepreneurial role models and college students’ entrepreneurial calling, took new exploration of the concepts and theories involved, and provided a new perspective for entrepreneurship education in colleges and universities.

## Data availability statement

The raw data supporting the conclusions of this article will be made available by the authors to qualified researchers upon reasonable request.

## Ethics statement

Ethical review and approval was not required for the study on human participants in accordance with the local legislation and institutional requirements. Written informed consent for participation was not required for this study in accordance with the national legislation and the institutional requirements.

## Author contributions

DJ, XL, and FZ wrote the manuscript and analyzed and interpreted the data. DJ and ZW made significant contributions to the conception and design of the work and to the revision of the manuscript. All authors contributed to this article and approved the submitted version.

## Conflict of interest

The authors declare that the research was conducted in the absence of any commercial or financial relationships that could be construed as a potential conflict of interest.

## Publisher’s note

All claims expressed in this article are solely those of the authors and do not necessarily represent those of their affiliated organizations, or those of the publisher, the editors and the reviewers. Any product that may be evaluated in this article, or claim that may be made by its manufacturer, is not guaranteed or endorsed by the publisher.
